# Supplementary material for: Coevolution of furA-Regulated Hyper-Inflammation and Mycobacterial Resistance to Oxidative Killing through Adaptation to Hydrogen Peroxide
Source: Microbiol Spectr. 2023 Jun 26;11(4):e05367-22. doi: 10.1128/spectrum.05367-22 (PMC10433983; doi:10.1128/spectrum.05367-22)
Supplement: Supplemental file 1 — Supplemental material. Download spectrum.05367-22-s0001.pdf, PDF file, 0.3 MB [file spectrum.05367-22-s0001.pdf]

Supplementary Information For

Co-evolution of *furA*-regulated hyper-inflammation and mycobacterial resistance to oxidative killing through adaptation to hydrogen peroxide

Xin Fan [a, †], Bei Zhao [b, †], Weishan Zhang [a, b], Ning Li [a], Kaixia Mi [a, b, #, *] and Beinan Wang [a, b, #, *]

[a] CAS Key Laboratory of Pathogenic Microbiology and Immunology, Institute of Microbiology, Chinese Academy of Sciences, Beijing, China.

[b] Savaid Medical School, University of Chinese Academy of Sciences, Beijing, China.

[†] These authors contributed equally to this work.

[#]Address correspondence to Beinan Wang, wangbn@im.ac.cn, Kaixia Mi, mik@im.ac.cn

[*] Present address: No. 1 Beichen West Road, Chaoyang District, Beijing 100101, China.

This PDF file includes:

Figure supplement 1

Figure supplement 2

Figure supplement 3

Table supplement 1

Table supplement 2

Fig. S1

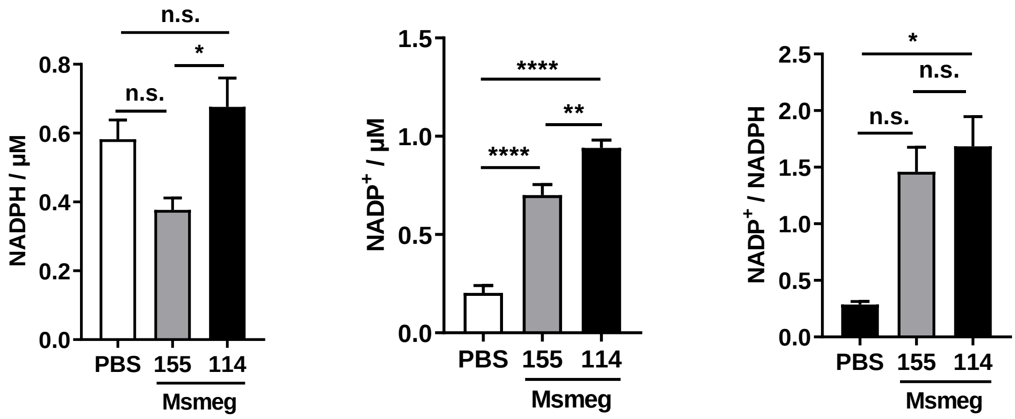

Fig. S1. Oxidative stress levels were detected in the single cell suspensions of lung tissue of mc^{2}114- or mc^{2}155-infected mice 24 h p.i. NADP$^+$ and NADPH levels were measured with a NADP$^+$/NADPH assay kit and NADP$^+$/NADPH ratio was calculated. Data are presented as means ± SEM from two independent experiments. Asterisks indicate significant differences (* P < 0.05, ** P < 0.01, **** P < 0.0001, and n.s., not significant by one-way ANOVA with Tukey’s test).

Measurement of NADP$^+$/NADPH Concentrations and Ratios

NADP$^+$ and NADPH was determined using a NADP$^+$/NADPH Assay Kit with WST-8 (S0179, Beyotime,China) according the manufacturer’s instructions as described previously (1). Briefly, the lung tissue samples were fixed in 3 mL of NADP$^+$/NADPH extract. The supernatants of lung tissue were prepared by mechanical dissociation of the tissue through a 70-μm nylon mesh and then centrifugation at 12000 rpm for 10 min at 4°C. Each of the supernatant samples was then separated into two portions. One portion was heated at 60°C to deplete NADP$^+$ (only NADPH left) while the other portion was left on ice as unheated sample (containing both NADP$^+$ and NADPH). NADP$^+$ could be reduced into NADPH in the

working buffer and the NADPH formed further reduced WST-8 to formazan. The orange product (formazan) was then measured at 450 nm spectrophotometrically. The $NADP^+$/NADPH ratio was calculated using following formula: (intensity of unheated sample – intensity of heated sample) / (intensity of heated sample). The results were normalized by protein concentration of each sample.

References

1. Wang Y, Jin H, Wang Y, Yao Y, Yang C, Meng J, Tan X, Nie Y, Xue L, Xu B, Zhao H, Wang F. 2021. Sult2b1 deficiency exacerbates ischemic stroke by promoting pro-inflammatory macrophage polarization in mice. Theranostics 11(20):10074-10090. https://doi.org/10.7150/thno.61646.

Fig. S2

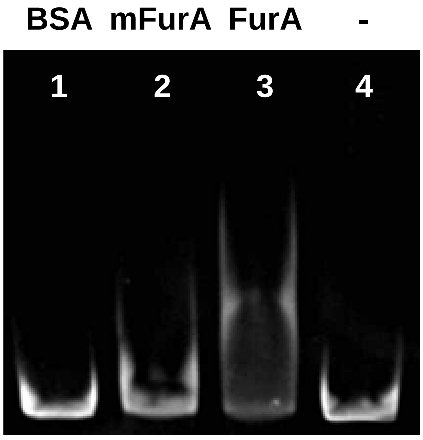

Fig. S2. Electrophoretic mobility shift assays (EMSA) were performed to assess the binding of mutated protein FurA to target DNA (the promoter region of *furA*). BSA, a negative protein; mFurA, a Fur protein from the mutated *furA* gene; FurA, a FurA protein from the wild type *furA* gene; -, no protein in the reaction.

Fig. S3

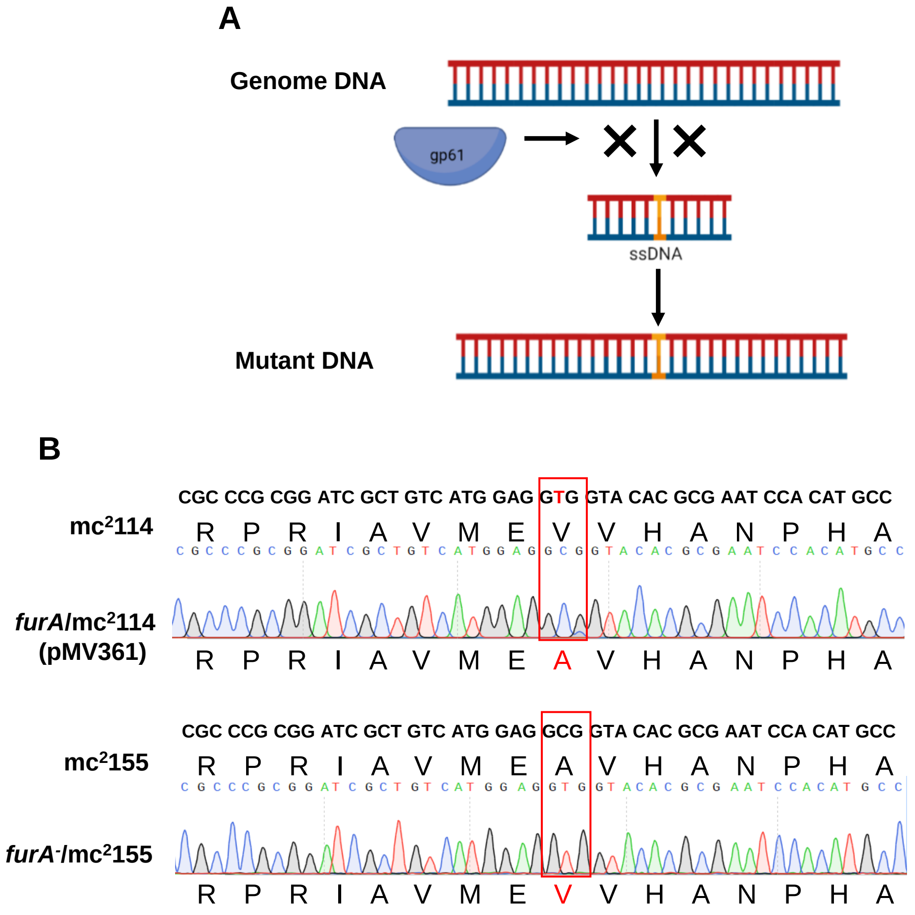

Fig. S3. Generation of *furA*/mc^{2}114 and *furA*$^-$/mc^{2}155 strains. (A) Schematic diagram showing the experimental design for producing a *furA* point mutation on the genome of mc^{2}155. The point mutation-containing ssDNA is recombined into the genome by the mycobacteriophage Che9C protein gp61. (B) The Sanger sequencing result of a single nuclear acid change in *furA/* mc^{2}114 and *furA*$^-$/mc^{2}155. The result showed that the nuclear acid T to C in *furA*/mc^{2}114 and C to T in *furA*$^-$/mc^{2}155.

Table S1. Mutations identified in mc^{2}114, compared to mc^{2}155

Mutation position	Gene	Protein	Reference nucleotide	Substitute nucleotide	Reference amino acid	Substitute amino acid
273886	MSMEI_0234	Transmembrane transport protein mmpL11	A	T	Y	*
436544			G	T		
663894			C	G		
663895			C	G		
663922			A	C		
2118639	MSMEI_1992	Flavin-binding monooxygenase	T	G	F	V
2464648			A	G		
2825972	MSMEI_2687	Conserved transmembrane alanine and leucine rich protein	A	G	T	T
3107175	MSMEI_2957	3-dehydroquinate synthase	G	A	G	R
3107182	MSMEI_2957	3-dehydroquinate synthase	G	A	G	D
3264632	MSMEI_3103	Maltooligosyltrehalose synthase	C	G	G	G
3503182	MSMEI_3351	Methyltransferase type 11	G	A	D	N
3528036			C	T		
3528131	MSMEI_3379	Ferric uptake regulator, Fur family	C	T	A	V
3530828			A	C		
4031782	MSMEI_3867	GntR family transcriptional regulator	T	C	V	V
4160153	MSMEI_3984	Putative monooxygenase	A	G	F	F
4213933			C	A		
4421749	MSMEI_4232	Glycerol-3-phosphate dehydrogenase GlpD1	T	C	G	G
4714916			A	C		
5585923			A	C		
5695012			T	A		
5695014			T	C		
5954735	MSMEI_5733	Conserved MCE-associated protein	G	C	Q	E
5981497	MSMEI_5760	Coenzyme F420-dependent oxidoreductase	T	C	D	G
6338150			T	G		
6338151			C	T		
6500474			A	C		
6725214	MSMEI_6490	Molybdate ABC transporter, permease protein	T	C	L	P

Table S2 Oligonucleotides used for generation of *fur*A point mutation in mc^{2}155

**Primers Name**	**5’-3’**
155-*furA*$^-$-Lag[a]	TCGTCTCGGTGTCGGCATGTGGATTCGCGTGTACCACCT CCATGACAGCGATCCGCGGGCGGGTGACCCGA
Hyg-Lag[a]	CAGAATTCCTGGTCGTTCCGCAGGCTCGCGTAGGAATCA TCCGAATCAATACGGTCGAGAAGTAACAGGGATTCTT
furA-F	CGAGACATGCACCGACATAC
155-*furA*$^-$ -F	CGGATCGCTGTCATGGAGCT
furA-R	CAGAAAGCCATCCAGAGCGT

[a] The point mutation sequences are shown underlined.
